# Mass Spectrometry Imaging of Lipids in Human Skin Disease Model Hidradenitis Suppurativa by Laser Desorption Ionization from Silicon Nanopost Arrays

**DOI:** 10.1038/s41598-019-53938-0

**Published:** 2019-11-25

**Authors:** Jarod A. Fincher, Derek R. Jones, Andrew R. Korte, Jacqueline E. Dyer, Paola Parlanti, Anastas Popratiloff, Christine A. Brantner, Nicholas J. Morris, Russell K. Pirlo, Victoria K. Shanmugam, Akos Vertes

**Affiliations:** 1 Department of Chemistry, George Washington University, Washington, DC 20052 USA; 2 Division of Rheumatology, George Washington University, Washington, DC 20037 USA; 3 Nanofabrication and Imaging Center, George Washington University, Washington, DC 20052 USA; 4 UES, Inc, Beavercreek, OH 45432 USA; 5 Chemistry Division, U.S. Naval Research Laboratory, Washington, DC 20375 USA

**Keywords:** Imaging studies, Mass spectrometry, Medical and clinical diagnostics

## Abstract

Neutral lipids have been implicated in a host of potentially debilitating human diseases, such as heart disease, type-2 diabetes, and metabolic syndrome. Matrix-assisted laser desorption ionization (MALDI), the method-of-choice for mass spectrometry imaging (MSI), has led to remarkable success in imaging several lipid classes from biological tissue sections. However, due to ion suppression by phospholipids, MALDI has limited ability to efficiently ionize and image neutral lipids, such as triglycerides (TGs). To help overcome this obstacle, we have utilized silicon nanopost arrays (NAPA), a matrix-free laser desorption ionization (LDI) platform. Hidradenitis suppurativa (HS) is a chronic, recurrent inflammatory skin disease of the apocrine sweat glands. The ability of NAPA to efficiently ionize lipids is exploited in the analysis of human skin samples from sufferers of HS. Ionization by LDI from NAPA allows for the detection and imaging of a number of neutral lipid species, including TGs comprised of shorter, odd-chain fatty acids, which strongly suggests an increased bacterial load within the host tissue, as well as hexosylceramides (HexCers) and galabiosyl-/lactosylceramides that appear to be correlated with the presence of HS. Our results demonstrate that NAPA-LDI-MSI is capable of imaging and potentially differentiating healthy and diseased human skin tissues based on changes in detected neutral lipid composition.

## Introduction

Lipids are a diverse class of biological compounds that play numerous roles in human physiology and disease. In addition to serving as the main building blocks of cell membranes, they are involved in a host of critical biological processes such as cell-to-cell signaling and energy storage^1^. Lipids constitute over half of the brain’s dry weight, and changes in certain lipid classes such as hexosylceramides (HexCers) and phosphatidylethanolamines (PEs) have been implicated in devastating neurological diseases, such as Alzheimer’s disease^2–5^. Neutral lipids, for example, triglycerides (TGs), diglycerides (DGs), and monoglycerides (MGs), steryl esters (SEs), and wax esters (WEs), are essential in energy storage but some of them, e.g., DGs, also function as second messengers in lipid signaling, and help to maintain sterol homeostasis (e.g., SEs)^6–8^.

With its ability to simultaneously provide detailed chemical information and reveal spatial distributions of analytes within tissue, mass spectrometry imaging (MSI) has the potential to provide significant insight into the roles of lipids in maintaining homeostasis and the development of disease. Imaging by mass spectrometry is an analytical technique that can detect and identify biomolecules with high chemical specificity, while simultaneously mapping their spatial distributions within biological tissue sections. Driven by this unique set of capabilities, MSI has seen widespread growth in fields such as the pharmaceutical industry and clinical diagnostics^9–12^. Matrix-assisted laser desorption ionization (MALDI) and secondary ion mass spectrometry (SIMS) were the first MSI platforms to capture the distributions of diverse chemical species in biological tissues^13,14^. Since then, MALDI, in which a UV-absorbing matrix is deposited onto the sample to facilitate laser desorption ionization (LDI), has become the most extensively used MSI platform. The widespread adoption of MALDI-MSI can be attributed in large part to the wide array of possible matrices, many of which provide selective ionization of certain compounds or classes of compounds^9,15,16^. This versatility has allowed for the imaging of a broad range of biomolecules including proteins, peptides, metabolites, and lipids^17–21^.

Despite the rapid advancements in the application of MALDI for MSI and the development of novel MALDI matrices, many challenges remain. Notably, MSI of neutral lipids, such as TGs, has been difficult using organic MALDI matrices, for example, 2,5-dihydroxybenzoic acid (DHB), due to ion suppression by phospholipids, e.g., phosphatidylcholines (PCs). To overcome this limitation, several novel methods, incorporating metal nanoparticles in place of organic matrices, have been developed facilitating the detection and imaging of TGs in the presence of PCs^22–24^. While these modifications have helped to extend the attainable lipid coverage in MALDI-MSI experiments, they still require deposition of a UV-absorbing material onto the tissue for MSI. This process can lead to inhomogeneous matrix deposition, diffusion of analytes, and creation of so-called “hot spots”, which negatively affect the achievable spatial resolution and can disrupt localization of analytes^15,25–27^.

Since the emergence of MALDI-MSI, several innovative matrix-free MSI platforms such as nanostructure-initiator mass spectrometry (NIMS), desorption/ionization on silicon (DIOS), desorption electrospray ionization (DESI), and laser ablation electrospray ionization (LAESI) have been developed. These techniques were developed for or adapted to imaging with the goals of circumventing the low mass interference introduced by the MALDI matrix, minimizing sample preparation, and extending the attainable molecular coverage for MSI applications^28–36^. Another matrix-free platform capable of MSI, LDI from silicon nanopost arrays (NAPA), was developed in our lab. It has been shown to provide ultra-trace sensitivity and the detection of a wide range of compounds and compound classes^37–40^. Previous MSI experiments examining mouse brain tissue sections on NAPA revealed enhanced ionization efficiency for certain lipid classes, such as HexCers and PEs, when compared to MALDI^41^. This enhanced sensitivity for difficult-to-detect species in tissues offers the possibility of using NAPA-MS to complement MALDI-MS for studying the roles of neutral lipids and other lipid classes in biological processes and disease.

For many years, antibiotics have been the first-line therapy for HS, however, routine cultures of HS lesions often fail to identify infection, or identify predominantly skin flora^42,43^. As genomic technologies have become more advanced, 16 S ribosomal RNA techniques have been used to study the HS microbiome and the mechanisms by which microbial dysbiosis contribute to disease development in HS^43^. Previous investigations of HS cases have established that patients have increased bacterial loads in affected areas, where it has been hypothesized that the infection is a result of the malfunctioning hair follicle and apocrine sweat gland region^44–47^. Anaerobes, in particular *Prevotella* (17.5%), *Porphyromonas* (6.2%), and *Fusobacterium* (10.3%), predominate in HS lesions^47^. When HS lesions become advanced, bacterial biofilms may contribute to disease pathogenesis, however, the mechanisms by which biofilms contribute to HS disease progression and the interactions between biofilms and the host immune response are poorly understood^48,49^.

The ability of NAPA-LDI-MSI to directly measure relevant chemical species with high spatial resolution makes it a promising platform for the study of host-pathogen interactions in systems such as HS-affected skin tissue. Here, we present the use of NAPA-LDI-MSI for imaging of neutral lipid distributions in human skin tissue. To illustrate potential medical applications, we apply NAPA-LDI-MSI to study lipid profiles and localizations in skin samples obtained from patients diagnosed with Hidradenitis suppurativa (HS), a chronic skin disease that causes inflammation and seriously compromises quality of life for many sufferers.

## Results

**Figure 1:**
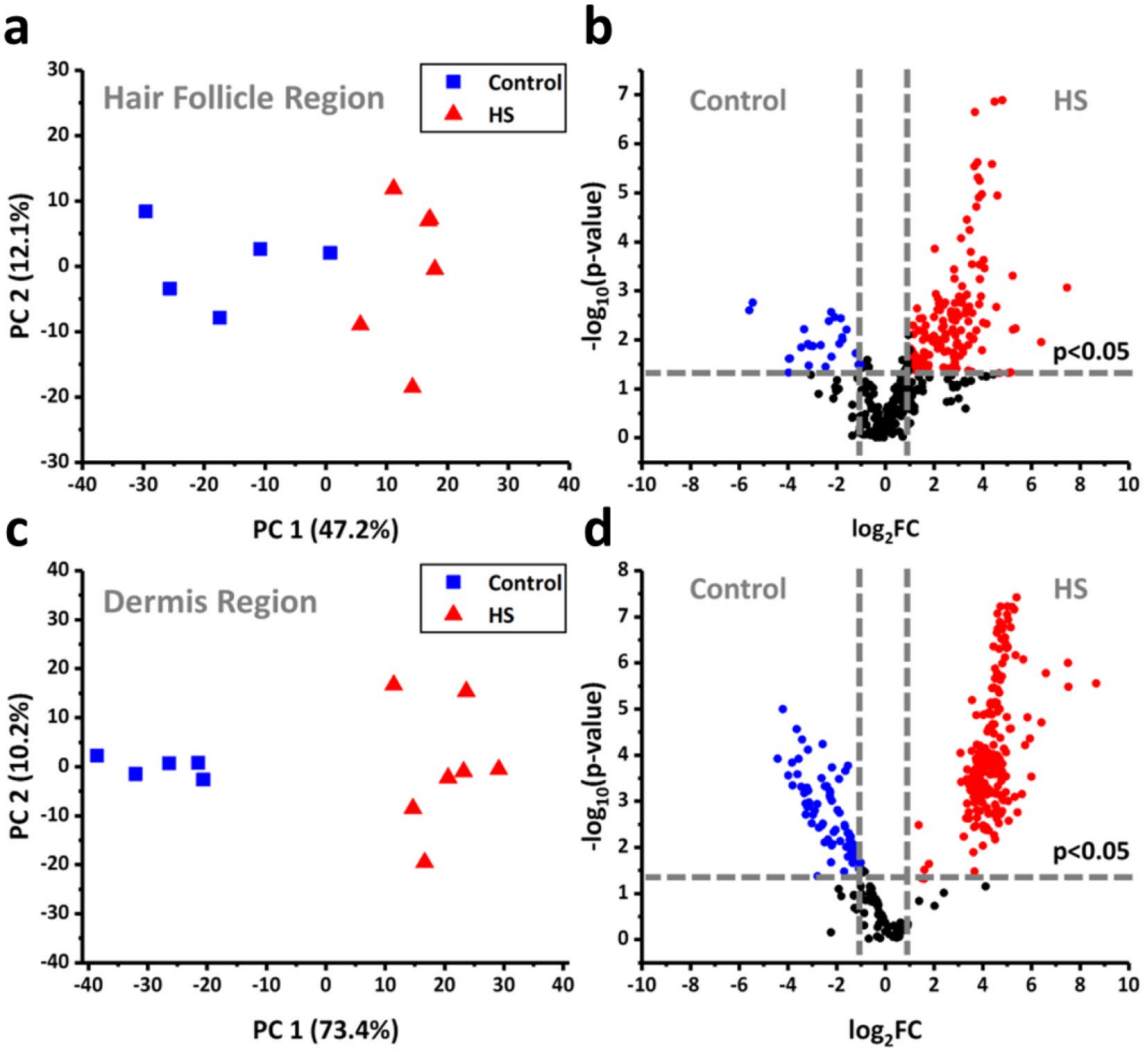
Principal component analysis scores plots for (**a**) hair follicle/apocrine sweat gland region and (**c**) dermis region. Volcano plots for (**b**) hair follicle region and (**d**) dermis region. Plots were produced by Origin 2019b.

To account for natural variation of lipid distributions within tissue sections, comparisons between control and Hidradenitis suppurativa tissues were constrained to two distinct regions identified by tissue morphology and confirmed by subsequently identified biomarkers. The first region used for comparisons consisted of the region surrounding the hair follicle and apocrine sweat gland, whereas the second region consisted of dermis tissue distant from these two features. Principal component analysis (PCA) of spectra obtained from the follicle/apocrine sweat gland region of control and HS skin sections allowed for the distinction of the two sample types (see the scores plot in Fig. 1a, and the loading plots in Supplementary Fig. S1 and Supplementary Table 1. Contributions of tentatively identified ions by lipid classes are color coded in the loading plots). A total of 170 ion signals were detected with significantly different ion intensities between HS and control tissues at fold-change, FC = I_HS_/I_C_, and p-value thresholds of FC > 2 or FC < 1/2 and p < 0.05, respectively (Fig. 1b). Subsequent identification of ion signals by accurate *m/z* search against a lipid database (LIPID MAPS; www.lipidmaps.org) and tandem MS analysis revealed a number of triglycerides (TGs) comprised of shorter, odd-chain fatty acids (C15:0, C13:0, etc.) to be significantly more abundant in HS tissue than the control (see Supplementary Table 2). These species exhibited fold increases in signal intensity ranging from 2 to 16 for HS tissue relative to the control. In contrast, TGs containing exclusively even-chain fatty acids (C16:0, C18:0, etc.) were detected more weakly in HS samples, with signal intensity fold decreases, −1/FC, ranging from −2 to −48 compared to control tissue (see Supplementary Table 2). Other tentatively assigned ions (i.e., from accurate *m/z* alone) representing neutral lipid classes, such as DGs, MGs, and WEs showed fold increases in signal intensity ranging from 13 to 37 for HS tissue compared to control.

Comparison of the dermis region of control and HS tissue analyzed by NAPA-LDI-MSI revealed even greater separation of the two tissue types by PCA (see the scores plot in Fig. 1c and the loadings in Supplementary Table 1). A total of 350 ion signals were detected with significantly different intensities between HS and control tissues at the same significance criteria (Fig. 1d). Subsequent identification revealed several members of another class of neutral lipids, hexosylceramides (HexCers) and galabiosyl-/lactosylceramides, to contribute significantly to the difference between the two tissue types. For HS diseased tissue, a statistically significant increase in the detection of galabiosyl-/lactosylceramides was observed within the dermis, with average fold increases ranging from 29 to 403. As with the hair follicle/apocrine sweat gland region, the lipids decreased in the HS diseased tissue consisted largely of TGs containing exclusively even-chain fatty acids, with average fold decreases, −1/FC, ranging from −6 to −22. Additionally, a significant increase of the tentatively assigned lipid species lysophosphatidic acid (LPA) and sphingosine-1-phosphate (S1P) was observed in the dermis region of HS tissue, with an average fold increase ranging from 9 to 42.

**Figure 2:**
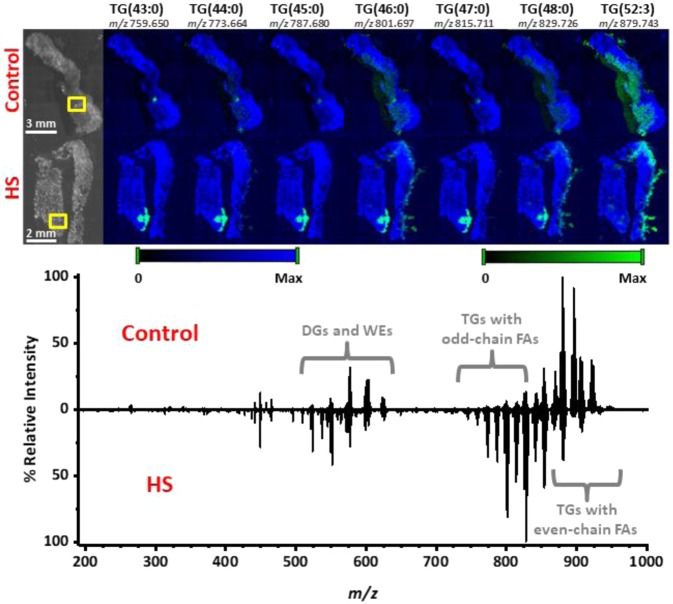
MS images acquired from 5 µm thick control (top row) and HS diseased (bottom row) tissue sections using NAPA-LDI-MSI. Chemical images (green channel) are overlaid onto a false-color representation of the optical image (blue channel). Ion intensity values were scaled equally between the two tissue types. Comparison of spectra obtained by averaging 8 MS scans from the hair follicle/apocrine sweat gland region (outlined in yellow boxes) of both tissue types. All TG lipids were detected as sodium adducts with a mass error of <5 mDa. Plots were produced by Origin 2019b.

**Figure 3:**
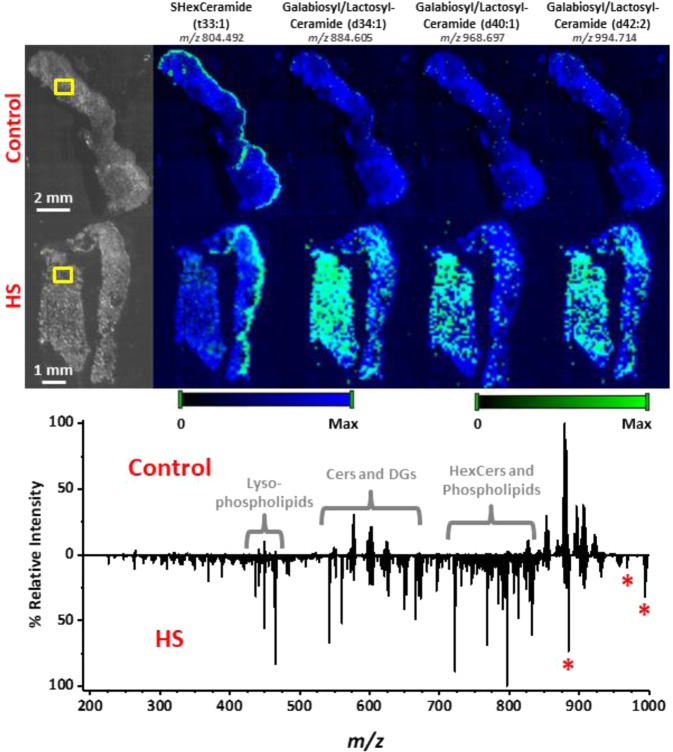
MSI analysis of 5 µm thick control (top row) and HS diseased (bottom row) tissue sections using NAPA-LDI-MSI. The selected chemical images show the spatial distributions of detected galabiosyl-/lactosylceramides (denoted by red asterisks), which were shown statistically to be present at remarkably higher levels in the HS diseased tissue. Chemical images (green channel) are overlaid onto a false-color representation of the optical image (blue channel). To help with visual orientation of the tissue sections, a chemical image of m/z 804.492, detected along the epidermis and tentatively assigned as SHexCer (t33:1) based on accurate *m/z*, is included. Comparison of spectra obtained by averaging 8 MS scans from the dermis region of both tissue types (outlined in yellow boxes). Ion intensity values were scaled equally between the two tissue types. All lipids were detected as sodium adducts with a mass error of <5 mDa. Plots were produced by Origin 2019b.

Following identification of species contributing to differences between HS and control tissues, MS images were generated to examine the distribution of these species within the tissue samples. Figure 2 presents the spatial distributions of several TGs varying by one carbon. TGs containing odd-carbon or short-chain fatty acids (43:0, 44:0, 45:0, and 47:0) were predominantly localized to the hair follicle/apocrine sweat gland region. Sample tandem MS spectra used for identification of these species are presented in Supplementary Fig. S2. Those containing longer-chain, even-carbon fatty acids were distributed more uniformly throughout the tissue. When comparing mass spectra (see Fig. 2), TGs consisting of shorter, odd-chain fatty acids found in the *m/z* 700–815 range were found to dominate the spectrum for HS tissue, whereas TGs consisting of longer, even-chain fatty acids found in the *m/z* 820–950 range were found to dominate the spectrum from control tissue. Moreover, MS images for one sulfatide (SHexCer) and several galabiosyl-/lactosylceramides can be found in Fig. 3. The galabiosyl-/lactosylceramides in particular were detected throughout the epidermis of HS tissues and were almost completely absent in control samples. This was further illustrated when comparing the mass spectra, as lipid classes such as galabiosyl-/lactosylceramides, HexCers, and phospholipids dominated the mass spectrum for HS diseased tissue, whereas native-like TGs consisting of longer, even-chain fatty acids dominated the control spectrum. Sample tandem MS spectra used for identification of these species are presented in Supplementary Fig. S3.

## Discussion

**Figure 4:**
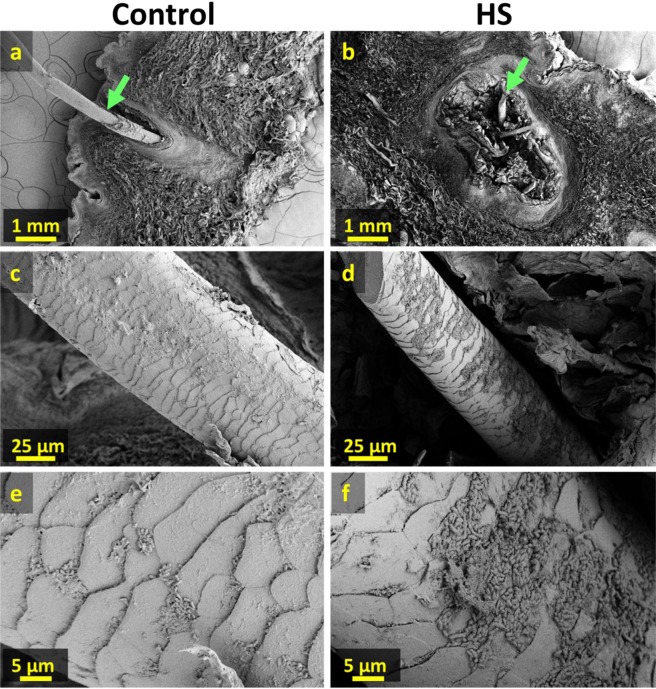
Scanning electron microscope images of control and HS affected tissue sections. (**a**) and (**b**) Cross sections of 300 µm thick control and HS affected tissue sections with hair follicle. (**c,d**) Higher magnification images of hair shaft (green arrows in (**a,b**)). (**e,f**) Higher magnification images showing increased presence of bacteria with emerging plaques along hair shaft in HS affected tissue.

**Figure 5:**
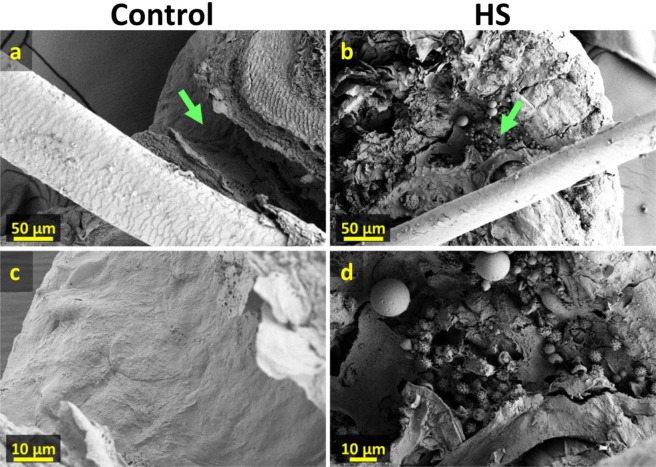
Scanning electron microscope images of control and HS affected tissue sections. (**a,b**) Cross section of 300 µm thick control and HS affected tissue sections with hair protruding through epidermis. (**c,d**) Higher magnification images showing the absence and presence of what appear to be immune cells along the epidermal surface (green arrows in (**a,b**)) for control and HS affected tissues, respectively.

Based on the ability of mapping neutral lipid distributions in human tissue by NAPA-LDI-MSI, our results help explore the localization of bacteria in HS affected skin. This is possible given that bacteria have been shown to be the primary source of odd-carbon number and short-chain fatty acids in mammalian tissues, where they are not natively produced in significant quantities^50–56^. We therefore hypothesize that the increased abundance of these lipids observed in HS-infected tissue is a result of increased bacterial load. Moreover, the apparently localized accumulation of these lipids (2 to 16-fold increase in HS near the hair follicles relative to control) is consistent with this observation. To confirm the localized increase in bacterial load, HS and control tissue samples (Fig. 4) were examined by scanning electron microscopy (SEM). In the control tissue sample (Fig. 4c,e), small colonies of bacteria were visible along the hair shaft. Given the ubiquity of bacteria on human skin and the critical roles the skin microflora plays in maintaining overall health, this finding was expected. In contrast, the hair shaft of the HS diseased tissue (Fig. 4d,f) showed a substantially greater number of bacteria forming contiguous sheets along the hair follicle. Moreover, significant accumulation of what appear to be immune cells (Fig. 5) was observed in the epidermis of HS diseased tissue (Fig. 5d), whereas they were absent in the control (Fig. 5c), indicating an immune response from the host tissue to bacterial infection. SEM imaging of tissue punches from HS diseased tissue also revealed a characteristic fistula, a hallmark of HS (See Supplementary Fig. S4b).

The significantly higher levels of galabiosyl-/lactosylceramides detected from HS diseased tissue by NAPA-LDI-MSI, as well as the apparent accumulation of immune cells in the HS diseased epidermis, support previous findings that accumulation of bacteria is associated with HS. Sphingolipids, for example galabiosyl-/lactosylceramides, are a class of neutral lipids that have been shown to be involved in a host of physiological processes, such as cellular stress response, apoptosis, cell proliferation, and microbial pathogenesis^57–60^. Certain sphingolipids have been shown to possess antibacterial activity toward specific bacterial strains, contributing to defense against pathogenic bacteria^61–65^. Furthermore, glycosphingolipids, such as lactosylceramides (LacCers), have become an area of research interest, as they have been shown to be involved in mediating innate immune responses, host-tissue pathogen interactions, and chronic inflammatory diseases^66–68^. The ability of LacCers to form “lipid rafts” within the cell membrane of host-tissue neutrophils confers binding selectivity against certain strains of bacteria, helping to target harmful pathogens^69^. The significant increase in galabiosyl-/lactosylceramides observed here is in agreement with the finding that enzymes responsible for generation of galabiosylceramides were significantly elevated in HS patients, although the isomeric galabiosyl- and lactosylceramides could not be distinguished in this work^70^. Lastly, the significantly increased intensity of ions tentatively assigned as LPA and S1P, both previously shown to help regulate immunity, cindicates the potential of using this technique for further study into the dynamics of the host tissue-pathogen interaction^71,72^.

As a matrix-free LDI-MSI platform offering enhanced ionization efficiency for classes of neutral lipids, e.g., TGs and sphingolipids, NAPA structures expand the coverage of lipid classes present in biological tissues. Serving as a complementary MSI platform, NAPA can be used in conjunction with MALDI, whereby imaging of different lipid classes from serial tissue sections can provide further insight into human diseases, such as heart disease and metabolic syndrome, as well as host-pathogen interactions. Given that neutral lipids have become a growing area of interest in biomedical research, the ability to detect and spatially map their distributions in biological tissues contributes to advancing this burgeoning field.

## Conclusion

Here we have shown an application of the NAPA-LDI-MSI platform to analyze and differentiate between control and HS diseased human skin tissue samples through differences in the observed lipid composition. The ability of NAPA to selectively ionize neutral lipid classes, such as TGs and galabiosyl-/lactosylceramides—lipid species that serve critical functions in numerous biological processes and are often difficult to detect in conventional MALDI-MSI—presents the possibility of using this platform to further investigate host-pathogen relationships, the process of pathogenesis, and defense mechanisms against infection.

## Methods

### Chemicals and materials

LC-MS grade water (catalog no. W6–212) and chloroform (catalog no. C6704–4) were purchased from Fisher Scientific (Hampton, NH). Carboxymethylcellulose (CMC; catalog no. C4888) was purchased from Sigma-Aldrich (St. Louis, MO). Lipid standard D-lactosyl-ß-1,1′ N-palmitoyl-D-erythro-sphingosine (LacCer(d34:1), catalog no. 860576 P) was purchased from Avanti Polar Lipids, Inc. (Alabaster, AL). SEM reagents glutaraldehyde (catalog no. 16020), paraformaldehyde (catalog no. 15710), sodium cacodylate buffer (catalog no. 11652), anhydrous ethanol (catalog no. 15055), and osmium tetroxide (catalog no. 19170) were all purchased from Electron Microscopy Sciences (Hatfield, PA). Conductive silver paint (catalog no.16062) was purchased from Ted Pella, Inc. (Redding, CA).

### Fabrication of NAPA imaging chips

The complete fabrication process for production of NAPA imaging chips has been previously described^73^. Briefly, NAPA imaging wafers were produced from low resistivity p-type silicon wafers using deep UV projection lithography (DUV-PL) followed by deep reactive ion etching (DRIE). Final dimensions for nanoposts were 1100 nm in height and 150 nm in diameter, with a periodicity of 337 nm.

### Tissue sample prep for MSI

Human skin tissues were harvested from four patients diagnosed with Hurley Stage III HS (diseased tissue) and three patients undergoing abdominoplasty (control tissue) in accordance with protocols approved by the GW Institutional Review Board 041408 and 101419, respectively. All human subjects gave written informed consent for collection of specimens and data, and all methods were performed in accordance with the relevant guidelines and regulations. Tissue samples were collected from the operating room immediately after being excised from the patient and washed with PBS solution before being stored at -80 °C. For cryosectioning, tissue samples were embedded in a 2.5% CMC solution and sectioned at a 5 µm thickness at -25 °C. Tissue sections were then thaw-mounted onto NAPA imaging chips and placed in a desiccator for ~30 min before MSI analysis.

### MSI data acquisition and image processing

All MSI analysis of human skin samples on NAPA was performed using a MALDI-LTQ-Orbitrap XL mass spectrometer (Thermo Scientific, San Jose, CA). A nitrogen laser emitting radiation at 337 nm with a focal spot size of ~100 µm × 80 µm was operated at a laser fluence of ~150 mJ/cm^2^ with 3 laser shots/scan and a raster step size of 100 µm. All mass spectra were acquired from *m/z* 180 and 1,000 using the orbitrap mass analyzer at a mass resolving power setting of 30,000. After acquisition, all MS images were generated with a *m/z* tolerance of 5 mDa using the ImageQuest software package (Thermo Scientific). Ion images were smoothed using the linear function.

### Scanning electron microscopy

Tissue sections and punches were fixed in a 2.5% glutaraldehyde, 1.5% paraformaldehyde, and 0.12 M sodium cacodylate buffer (pH 7.4) solution for a minimum of 24 h, then rinsed with a 0.12 M sodium cacodylate buffer (pH 7.4) at 3 × 15 min intervals and placed in a 1% OsO_4_ solution for 1 h. Samples were then dehydrated by subsequent immersion in 9 ethanol-water solutions with increasing concentration (15% up to 100%) at 15 min intervals before immediately being placed in a Tousimis 931 critical point dryer (Tousimis, Rockville, MD). After fixation, samples were mounted onto SEM stubs and sputter coated (Cressington Scientific Instruments, Watford, England) with a 6 nm-thick coating of iridium. All SEM images were acquired using a FEI Teneo LV FEG SEM (Thermo Fisher Scientific, Waltham, MA) with a high voltage of 2 kV and a current of 13 pA.

### Data analysis

After acquisition, raw data files (*.raw) were imported into ImageQuest (Thermo Scientific, San Jose, CA) for processing. MS scans from the hair follicle/apocrine sweat gland region and dermis region of both tissue types were averaged and exported, then uploaded into mMass for peak picking and deisotoping^74^. Principal component analysis was performed using MetaboAnalyst (https://www.metaboanalyst.ca; last visited 12/28/2018). Parameters for performing PCA analysis of the hair follicle/apocrine sweat gland region and dermis region consisted of a 7 mDa mass window for binning of mass spectra, as well as a setting of 65% and 67% missing values threshold, respectively. Furthermore, all data were normalized to the sum, log transformed, and scaled using Pareto scaling. The *m/z* values identified as statistically significant (p < 0.05) with a signal intensity fold-change between diseased and control greater than 2 were compared against the LipidMaps reference database, (http://www.lipidmaps.org/), for tentative lipid identifications based on mass accuracy (±5 mDa). Finally, confirmation of lipid identifications were carried out using tandem MS with fragmentation induced by collisional induced dissociation (CID). TGs were identified based on observed neutral fatty acid chain losses, whereas galabiosyl-/lactosylceramides were identified based on individual sugar losses, and by spectral comparison to a commercial standard.

## Supplementary material

Supplementary Information

Supplementary Information

Supplementary Information
