# Supplementary material for: Mass Spectrometry Imaging of Lipids in Human Skin Disease Model Hidradenitis Suppurativa by Laser Desorption Ionization from Silicon Nanopost Arrays
Source: Sci Rep. 2019 Nov 25;9:17508. doi: 10.1038/s41598-019-53938-0 (PMC6877612; doi:10.1038/s41598-019-53938-0)
Supplement: Supplementary file 1 — Supplementary Information [file 41598_2019_53938_MOESM1_ESM.docx]

***Supplementary Information for***

**Mass Spectrometry Imaging of Lipids in Human Skin Disease Model Hidradenitis Suppurativa by Laser Desorption Ionization from Silicon Nanopost Arrays**

Jarod A. Fincher,^1^ Derek R. Jones,^2^ Andrew R. Korte,^1^ Jacqueline E. Dyer,^1^ Paola Parlanti,^3^

Anastas Popratiloff,^3^ Christine A. Brantner,^3^ Nicholas J. Morris,^4^ Russell K. Pirlo,^5^ Victoria K. Shanmugam,^2^

and Akos Vertes*^1^

*^1^Department of Chemistry, George Washington University, Washington, DC 20052, USA*

*^2^Division of Rheumatology, George Washington University, Washington, DC 20037, USA*

*^3^Nanofabrication and Imaging Center, George Washington University, Washington, DC 20052, USA*

*^4^UES, Inc., Beavercreek, OH 45432, USA*

*^5^Chemistry Division, U.S. Naval Research Laboratory, Washington, DC 20375, USA*

*Correspondence should be addressed to A.V. (email: vertes@gwu.edu)

| 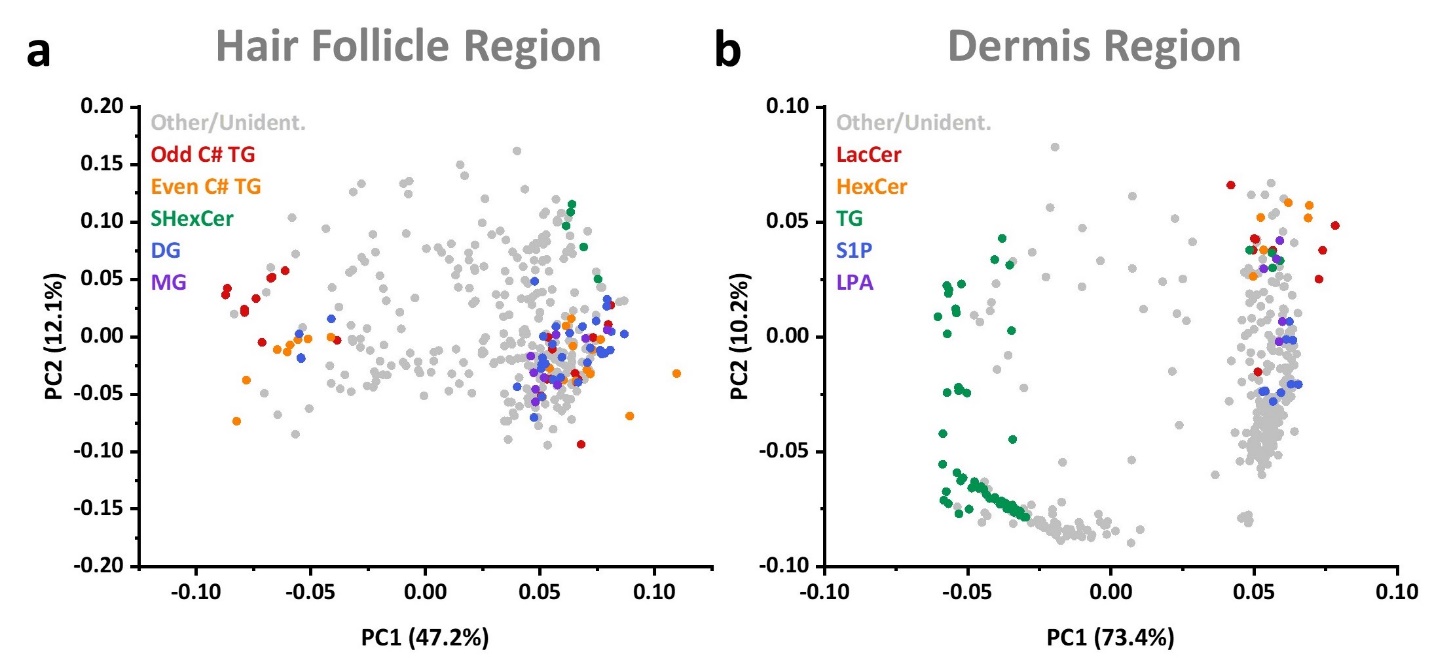 |
| --- |
| **Supplementary Figure S1:** Principal component analysis loadings for comparison of HS-affected and control skin samples in a) hair follicle region and b) dermis region. Contributions of tentatively identified ions by lipid classes are color coded in the loading plots. For ions with potential IDs belonging to more than one lipid class (here, TGs, DGs, and/or MGs), the more highly substituted class was displayed (TG>DG>MG). |

| 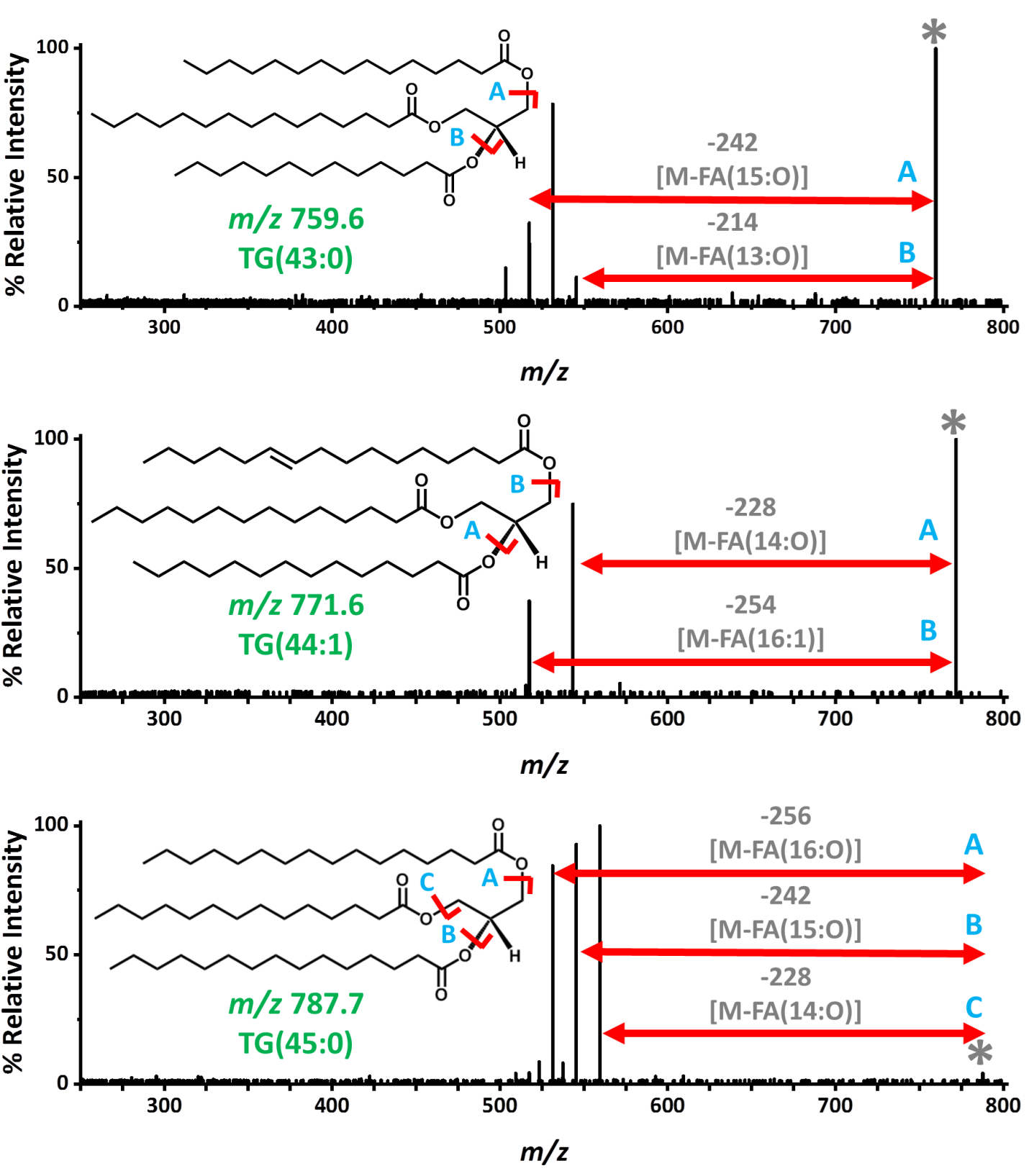 |
| --- |
| **Supplementary Figure S2:** Tandem mass spectra of *m/z* 759.6 TG(43:0), *m/z* 771.6 TG(44:1) and *m/z* 787.7 TG(45:0). Based on FA losses, these TGs are identified as TG(15:0/15:0/13:0), TG(14:0/14:0/16:1), and TG(16:0/15:0/14:0) and their isomers, respectively. All three TG lipid species were detected as sodiated adducts. |

| 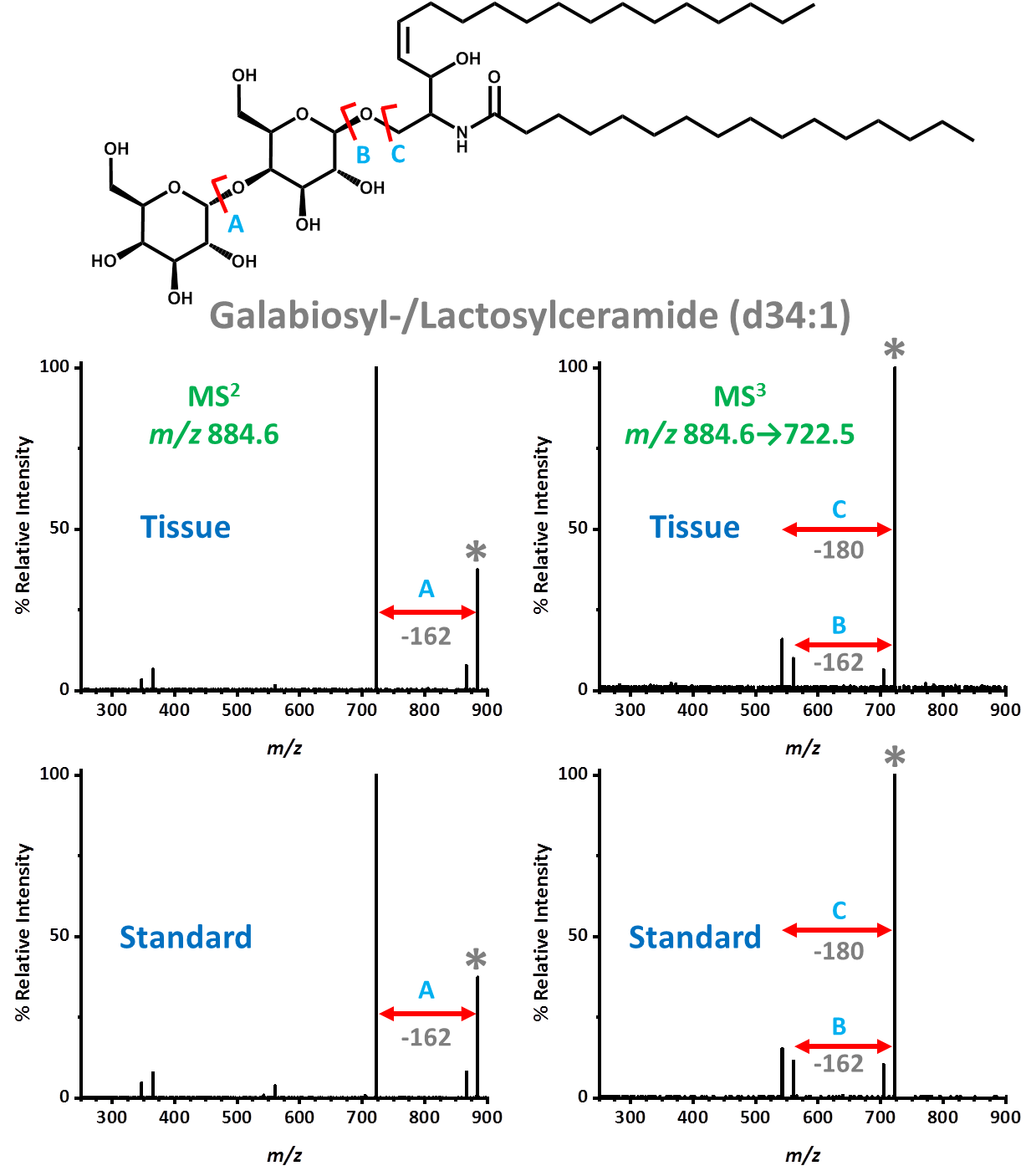 |
| --- |
| **Supplementary Figure S3:** MS^2^ and MS^3^ of m/z 884.6, identified as sodiated galabiosyl-/lactosylceramide (d34:1). |

| 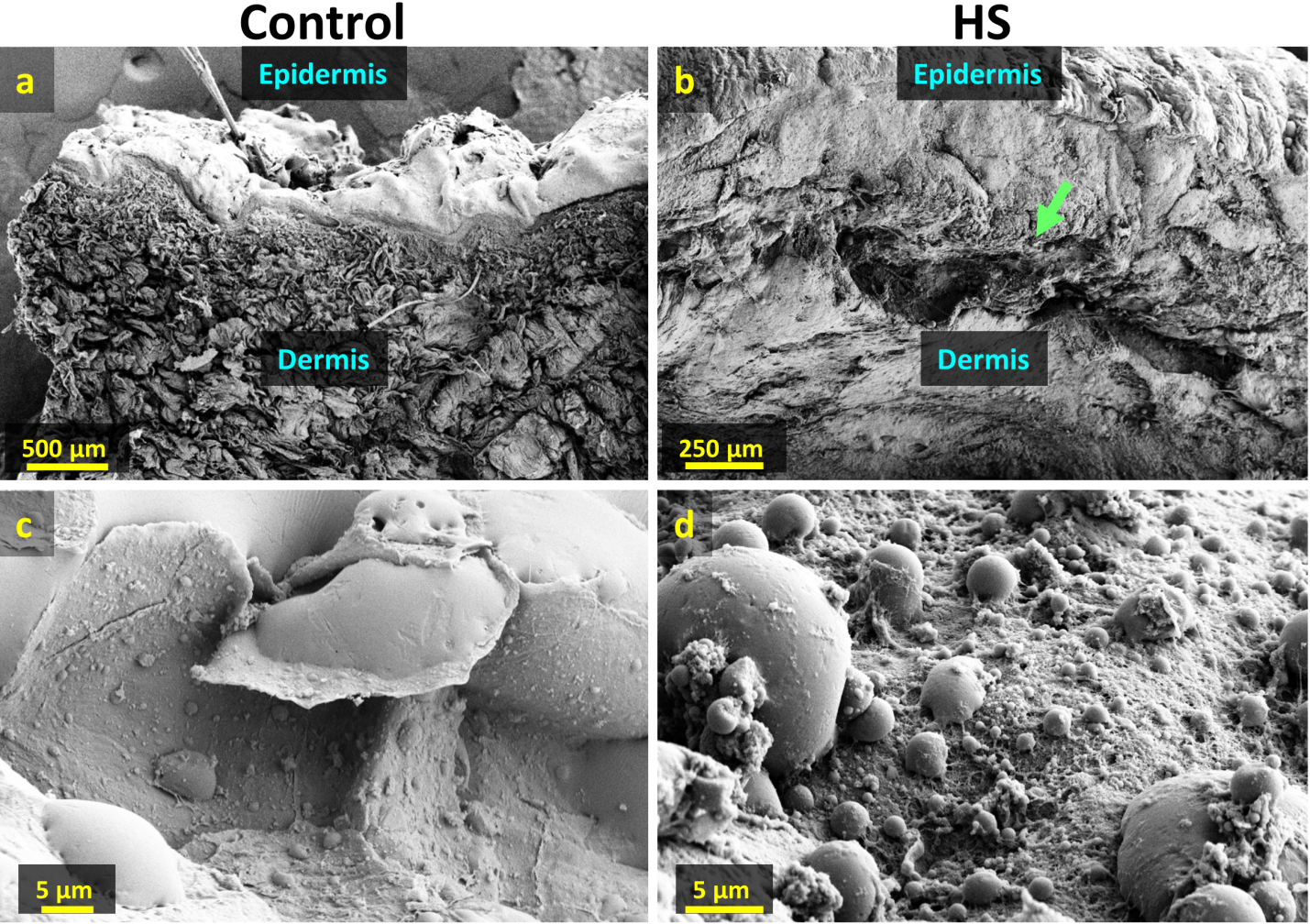 |
| --- |
| **Supplementary Figure S4:** Scanning electron microscope images of control and HS diseased skin tissue. a) Cross-sectional view of control skin tissue. b) Cross-sectional view of HS skin tissue, where the characteristic “tunnel-like tract” (denoted by green arrow) can be seen in the dermis region. c) and d) Epidermis surface (top-down view) for control and HS diseased tissue, respectively. |
